# Social and Poor-Law Problems

**Published:** 1906-07-21

**Authors:** 


### SOCIAL AND POOR LAW PROBLEMS.

### THE BUSINESS OF BEING A GUARDIAN.

The revelations which are being made at the Local Government Board inquiries at Poplar and West Ham will doubtless have a salutary effect for the moment in checking the extravagance which has of late years been introduced into Poor-law administration, largely through the influence of sentimental enthusiasts, who have, though quite unconsciously, played into the hands of the dishonest among Guardians and contractors and the disreputable among the poor. Unfortunately, another result will be to make the title of Guardian a by-word, a synonym for dishonesty, self-indulgence, and general inefficiency. And unhappily the small esteem in which the office of Guardian will be held will make it more difficult than ever to get the right people to serve in that capacity.

As it is, the position is a thankless one. There is no pay attached to it, and very few perquisites. Few people have the least idea of the time occupied by the proper fulfilment of a Guardian's duties, and still fewer realise the various kinds of knowledge requisite for it. It is not merely the dealing with all the complicated problems of poverty, though that is a responsibility serious enough, and demands a knowledge of history and economics which it would be rash to predicate of any one chosen by popular election. But when one has to make contracts for the feeding, clothing, and housing of thousands of people; when one is responsible for the selecting of officers who are to deal with these thousands in every crisis of life—birth, education, apprenticeship, unemployment, sickness, death—it is obvious that the ideal guardian should possess encyclopædic knowledge and all but omniscient wisdom. And the position is, in nine elections out of ten, left to go by default to any one who chooses to accept it—to a small tradesman who hopes to make something, directly or indirectly, out of it; to a trades-union leader whose sole aim is to keep up the price of any work done for the Union, and if possible to hinder the acceptance of tenders except from certain favoured firms; to a sentimental lady who is inclined to carry out in poor-law work the same methods which make her the prey of the loafer in private charity; or to some business man, who, having retired from active work, takes up the guardianship as an amusement.

This last is, if he is not too old to have lost his insight and energy, admirably fitted for the post, but the "if" represents a very great risk. Nor is the tradesman unsuitable, if one could be sure of his probity and unselfishness. Among the constituent members of a Board of Guardians there should certainly be the "butcher and baker and candlestick-maker" or his modern equivalent, the electrician. One would add to these a plumber, a grocer, a draper, an upholsterer, and a builder. It is true that there is always a steward to look after consumable stores, and usually a clerk of works to supervise building and the repairing of buildings. But unless there is some supervision of these officials, they will certainly become the Guardians' masters instead of their servants, and will be open to temptations to carelessness and extravagance, if not of actual dishonesty. In the matter of contracts, it is most desirable that expert knowledge should be available. It is safe to assume that of every article contracted for a large proportion of the Guardians know absolutely nothing. This is no reflection on any. Why should the draper Guardian be expected to understand the contract price of soap, or the grocer that of calico? Every Guardian votes as to the giving of a contract, but all must, or ought to be, guided by the opinion of those who have a practical knowledge of the article being dealt with. The instinct of the non-expert is simply to vote for the lowest tender, but this instinct may be modified by the opinion of an expert as to quality. Both considerations are, however, often overruled by a consideration of a very different nature—local influence. The preference to a local man is not always dishonest. "If we give a local man the contract," it is argued, "we are enabling him to give work to the people of our own parish, and it is as wise to give them employment outside the workhouse as to maintain them in it." A perfectly reasonable argument if human nature were invariably pure and incorruptible, but it is apt to end, as we all know, in the local man getting, not a reasonable preference, but an unreasonable monopoly at any terms he chooses to make. Experience of this kind of thing drives honest people back to the hard rule—merciless but just—accept the lowest tender, and if it is found that the contractor does not supply goods up to specification, break the contract without hesitation. But in such crises the advice of the expert again is needed, lest the steward, or whoever has charge of the article in question, is tempted, through bribery or friendship, to make unreasonable complaints. From this slight sketch of a Guardian's responsibilities, it will be seen that the post ought to be occupied by energetic, capable men, in close touch with some kind of business bearing on the matters that will come within their ken on the Board. But such men are, as a rule, fully occupied with their own affairs. They cannot give the better part of three, four, and sometimes even five days a week to Poor-law work without neglecting their own interests.

Then there is the supervision of the administration. Not merely the regular, periodic inspection, for which the officials are prepared, and for which they have everything in order, but the visits without notice—the dropping into the workhouse at dinner-time to see how the food is served, and to sample it—the look into the wards of the infirmary to see if, when nobody is expected, the patients are clean and comfortable; into the schools to see if discipline is strict without being harsh. Add to this the visits that must be paid to lunatics at various asylums, to boarded-out children, to convalescents, and it will be seen that to do the work of a Guardian properly, man or woman must be possessed of not merely special knowledge of one department or another of the many subjects dealt with, but also of common sense, tact, energy, and abundant leisure. To say that all Guardians do not possess these qualifications is not to disparage them very much.

But it is this general lack of fitness for the post or of time to give to the work that is really mischievous—far more than the occasional personal unworthiness of an individual Guardian. It is not the rule in most Unions, whatever it may be in Poplar, for Guardians to get drunk at the ratepayers' expense. In many even the officials get no alcohol at all, and the inmates only the scanty ounces ordered by the doctor. In many Unions neither the Guardians nor their relations are interested in the contracts. But in all, contracts are voted on by people who know nothing of the value of the articles wanted, and in all there is a tendency to carelessness in the matter of expenditure, both in unconsidered generosity to the poor, and in thoughtless expenditure on buildings and administration. The remedy for this is the "surcharging" of the Guardians by the Local Government Board—that is, making them pay out of their own pockets for anything which that body considers unnecessary or extravagant. The Local Government Board does not apply this whip very freely, and perhaps some Boards of Guardians have forgotten its existence. In the immediate future it will probably be better remembered than it has been in the past, and will form a needed check to carelessness and extravagance.

